# A survey on stress, anxiety, depression and coping styles in dental undergraduates from 34 universities in China

**DOI:** 10.1186/s12909-026-09371-9

**Published:** 2026-05-09

**Authors:** Ying Fang, Jianwen Li, Mingya Yang, Yijing Zhang, Wenyan Huang, Si Meng, Xi Xiang, Guohou Miao, Sujuan Zeng

**Affiliations:** 1 Department of Pediatric dentistry, School and Hospital of Stomatology, Guangdong Engineering Research Center of Oral Restoration and Reconstruction & Guangzhou Key Laboratory of Basic and Applied Research of Oral Regenerative Medicine, Guangzhou Medical University, Guangzhou, 510182 China; 2 Department of School of Stomatology, Guangzhou Medical University, Guangzhou, 511436 China; 3 Department of School of Economics and Management, South China Agricultural University, Guangzhou, 510640 Guangdong China; 4 School and Hospital of Stomatology, Guangdong Engineering Research Center of Oral Restoration and Reconstruction& Guangzhou Key Laboratory of Basic and Applied Research of Oral Regenerative Medicine, Guangzhou Medical University, Guangzhou, 510182 China

**Keywords:** Dental Undergraduates, Depression-Anxiety-Stress, Adaptive Coping, Mental Health Disparities, Cross-Sectional Studies

## Abstract

**Background:**

Dental students face intense academic and clinical pressures, yet large-scale studies utilizing rigorous theoretical frameworks to evaluate their mental health remain limited in China.

**Objective:**

This study aimed to investigate the prevalence of depression, anxiety, and stress among Chinese undergraduate dental students, identify key sociodemographic and experiential correlates, and evaluate the associations of coping styles with mental health outcomes using a Directed Acyclic Graph (DAG) framework.

**Methods:**

Following STROBE guidelines, a national cross-sectional survey was conducted among 1,712 dental undergraduates from 34 universities across five geographic regions. Psychological distress was assessed via the DASS-21, and coping mechanisms via the SCSQ. Multivariable analyses were employed—specifically, multiple linear regression for continuous subscale scores and ordinal logistic regression for categorical severity levels—to identify factors independently associated with psychological morbidity.

**Results:**

The prevalence of at-risk symptoms was 46.1% for anxiety, 36.0% for depression, and 24.4% for stress. Significant regional disparities were observed, with students in the Western region reporting the highest distress levels (Mean total score = 17.81 ± 10.59; *p* < 0.001). In multivariable models, suboptimal physical health emerged as the strongest factor independently associated with distress (β = 0.37; AOR = 3.71–4.44). While positive coping exhibited a significant inverse association with symptom severity (AOR = 0.87–0.92, *p* < 0.001), negative coping, poor college adaptation (β = -0.15), appearance dissatisfaction (β = -0.13), and excessive family interference were associated with significantly increased psychological burden.

**Conclusion:**

Chinese dental undergraduates experience a substantial burden of psychological distress, characterized primarily by anxiety and depression. This distress is disproportionately distributed, with students in Western China and those reporting suboptimal physical health emerging as particularly vulnerable. Positive coping strategies act as vital buffers associated with better mental health, whereas negative coping, body image dissatisfaction, and poor college adaptation are linked to increased psychological burden. Dental education institutions must implement targeted, evidence-based psychological support systems prioritizing these vulnerable cohorts.

**Supplementary Information:**

The online version contains supplementary material available at 10.1186/s12909-026-09371-9.

## Introduction

According to the latest data from the World Health Organization (WHO), suicide ranks as the third leading cause of death among individuals aged 15–29, highlighting the severe mental health challenges confronting contemporary youth [1]. A comprehensive meta-analysis of psychological and behavioral symptoms in medical students globally has revealed that nearly one-third experience various forms of psychological distress, including stress, anxiety, and depression, with prevalence rates significantly higher than in the general population, warranting urgent scholarly attention [2]. Multiple studies have shown that medical students face significant academic, professional, and interpersonal pressures, which together contribute to a high prevalence of mental health problems [3, 4]. Studies have shown that medical students commonly experience fear of failure, doubts about personal competence, and stress regarding future uncertainty and high career expectations [5, 6]. The complexity and multidimensionality of these factors further exacerbate psychological stress among undergraduate dental students [7, 8].

Undergraduate dental students face multiple stressors. Previous studies have shown that the dental curriculum is highly integrated and complex, places strong emphasis on practical skill development [9], and is characterized by its continuous, long-term, and demanding nature [10–12]. In fact, a study of dental undergraduates in China found that academic stressors, such as worries about academic performance and a heavy curriculum, were the main source of students’ perceived stress [13]. Among graduating-year dental students in China, the prevalence of depression, anxiety, and stress has been reported as high as 28.8%, 32.9%, and 17.9%, respectively, providing concrete epidemiological evidence for the severity of the problem [14]. In addition, personal factors such as family and social support, financial pressure, and health status are closely related to their mental well-being [13, 15]. A study from Malaysia showed that good social support is an important protective factor against mental illness, whereas dental students tend to use negative ways of coping with stress such as alcohol, smoking or drugs [16]. It is worth pointing out that dental students do face tremendous stress, and these stresses place a heavy psychological burden on them and should be of concern to dental educators [17].

Amidst increasing higher education enrollment and imbalanced job markets, psychological morbidity among Chinese medical students has escalated significantly [18]. While this trend has been noted, research into regional disparities in the mental health status of dental students remains critically limited. Existing studies on medical students offer some clues: for example, a study of medical students in Inner Mongolia found that medical students in the region faced greater psychological stress and emotional difficulties, but the study sample was small and no comparisons were made with other regions [19]. While one investigation suggested Middle Eastern medical students exhibit the highest depression rates—followed by Chinese cohorts—this finding derived from only three international institutions, potentially misrepresenting China’s diverse contexts [20]. Dental-specific literature is even more scarce; although one study across seven European countries found that dental students from different nations reported psychological problems differently, possibly related to cultural backgrounds and educational systems [21], another study in Colombia found no significant domestic geographical disparities [22]. This scarcity of research, particularly in a vast and culturally diverse country like China, underscores a key gap this study aims to fill. Crucially, dental undergraduates’ psychological status directly influences academic performance and life quality [23, 24]. Thus, comprehensive analysis of stress-depression-anxiety determinants is essential for evidence-based educational reforms and student psychological burden mitigation.

The Depression, Anxiety and Stress Scale-21 (DASS-21), a globally validated abbreviated version of the 42-item DASS, quantifies subjects’ psychological distress across three domains [25]. While extensively applied in mainland Chinese university populations, evidence gaps persist regarding dental cohorts’ psychometric profiles using this instrument [26–28]. The Simplified Coping Style Questionnaire (SCSQ) is a shortened version of the Coping Style Questionnaire (CSQ) designed to assess coping outcome expectations, including positive, negative, or neutral responses. It has been found to correlate significantly with the traditional coping questionnaire and to capture the impact of socioeconomic status differences on self-reported health status [29]. In addition, the simplified questionnaire has shown the advantage of reducing nonresponse and increasing valid sample size in clinical trials [30].

Therefore, well-designed, nationwide studies are imperative to elucidate the complex interplay between demographic, academic, and psychosocial factors influencing the mental well-being of this vital student population. The present study was designed to address these gaps by investigating the prevalence and severity of depression, anxiety, and stress among Chinese dental undergraduates. The primary objectives were to identify key sociodemographic factors associated with psychological distress and to evaluate the associations between different coping styles and mental health outcomes. We hypothesized that: (1) psychological distress would vary significantly across different academic years and geographical regions; (2) students facing higher financial or academic burdens would report higher levels of stress and anxiety; and (3) adaptive coping strategies would be associated with more favorable psychological outcomes, while negative coping would show a reverse association. By situating these findings within the context of Chinese dental education, this study aims to provide a robust evidence base for the development of targeted, evidence-based psychological support systems.

## Materials and methods

### Study design and participants

This investigation constituted a national cross-sectional online survey designed to assess the mental health status of undergraduate dental students in mainland China. The study population comprised students officially enrolled in a stomatology (dentistry) program at universities across the country. Eligible participants were defined as all current undergraduate dental students, ranging from the first to the fifth (final) academic year. Postgraduate students (e.g., master’s or PhD candidates), advanced training students, and international students were excluded from this study.

A total of 1,712 valid responses were included in the final analysis. As this was a nationwide exploratory survey aiming to capture a wide spectrum of experiences from a geographically dispersed population, a purposive sampling strategy aimed at maximum variation was employed to ensure representation from diverse universities and regions. Consequently, a priori sample size calculation or power analysis was not performed, with the final sample size being determined by the number of complete responses received during the data collection window. Nevertheless, the final sample size of 1,712 is robust for the planned statistical analyses. According to the rule of thumb for multivariable regression, which suggests a minimum of 15–20 participants per predictor variable, our sample size far exceeds the requirement for the approximately 20 variables included in our models. Furthermore, based on an estimated total population of approximately 60,000 dental undergraduates in China, a sample of 1,712 provides a margin of error of approximately ± 2.32% at a 95% confidence level, ensuring high precision for the reported prevalence rates of depression, anxiety, and stress.

This study was conducted in accordance with the Declaration of Helsinki and was approved by the Ethics Committee of Guangzhou Medical University Dental Hospital (Ref. No.: LCYJ20250808003). Participation in the survey was entirely voluntary. Informed consent was obtained electronically from all participants prior to their involvement, where they were required to read a detailed information sheet and click an “I agree” button to proceed. The study was designed to be anonymous, with no personal identifiers collected during the data collection process. All data were stored securely on password-protected servers, and confidentiality was maintained throughout the research and publication process. The study design and reporting followed the Strengthening the Reporting of Observational Studies in Epidemiology (STROBE) reporting guideline for cross-sectional studies.

### Sampling strategy and data collection procedure

The sampling framework aimed to achieve a broad geographical distribution across the nation, acknowledging that strict national representativeness cannot be fully guaranteed with a convenience sampling approach. Based on information from the Ministry of Education and Chinese Stomatological Association, there are approximately 65 dental schools in mainland China. From this total, we purposively selected 34 universities to ensure representation from all five major geographical regions: Northern, Southern, Eastern, Central, and Western China. Specifically, the 34 institutions were selected from the initial pool of 65 dental schools based on their geographical location, school ranking, and willingness to collaborate. We initially invited 45 universities that met the regional balance criteria via formal email or professional liaison; 34 schools (75.6% response rate) agreed to participate and facilitated the distribution of the survey link to their respective undergraduate students. The primary sampling method was a combination of convenience and snowball sampling. The survey link was disseminated through the research team’s professional networks by contacting faculty members or student leaders at the target universities, who then distributed the link to class groups and student union social media channels within their institutions. Additionally, the questionnaire was posted on open, dental student-focused online forums to broaden the reach. Specifically, WeChat and QQ—the most widely used social media platforms among Chinese university students—were utilized as the primary vehicles for link dissemination. These links were precisely targeted to dental students by distributing them within verified major-specific class groups and institutional student union channels at each of the 34 participating universities.

Data were collected using the Wenjuanxing platform (https://www.wjx.cn/), a widely used online survey tool in China. The data collection period was from March to June 2025. Prior to accessing the questionnaire, all potential participants were presented with a detailed information sheet outlining the study’s purpose, procedures, and the voluntary and anonymous nature of participation. Informed consent was obtained electronically; only individuals who clicked the “I agree” button were permitted to proceed to the survey questions. To ensure data quality, the platform was configured to record response times, and a minimum completion time was set to discourage random responding. To verify eligibility, a mandatory screening question was included at the beginning of the survey, requiring participants to confirm their status as a current undergraduate dental student. Technical measures were further strengthened on the Wenjuanxing platform by restricting submissions to one per unique device ID and one per verified WeChat account, in addition to IP address tracking, to prevent duplicate responses. To minimize self-selection bias, the survey recruitment materials were framed broadly as a study on “student life and well-being” rather than focusing exclusively on “mental disorders,” thereby encouraging participation from students with varying psychological states. Furthermore, to mitigate non-response bias, follow-up reminders were sent through student leaders twice a week during the collection period to encourage participation from those who might have initially overlooked the invitation. Based on enrollment data provided by the coordinating faculty at the 34 participating schools, the total potential participant pool was estimated at approximately 5,000 students; thus, the 1,712 valid responses represent an estimated response rate of 34.2%.

### Recruitment and quality control

**Fig. 1:**
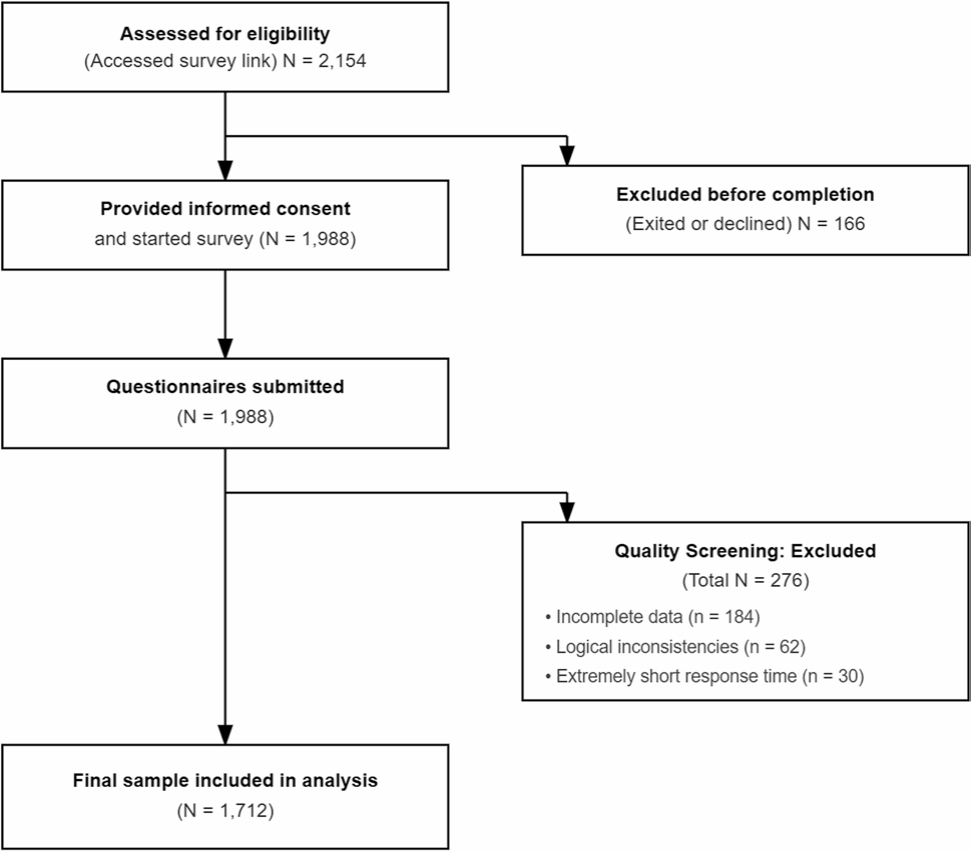
Participant flow diagram

The recruitment and screening process followed a rigorous funnel to ensure data integrity. A total of 2,154 students accessed the survey link during the recruitment period. Of these, 1,988 participants provided informed consent and initiated the survey. To ensure data rigor and minimize selection bias, questionnaires were excluded if they met any of the following criteria: (1) incomplete responses (missing more than 10% of items); (2) logical inconsistencies (e.g., age-grade mismatch); or (3) low-effort engagement (completion time less than 120 s). Following these screening procedures, 276 cases were excluded (184 incomplete, 62 logical errors, and 30 rapid completions), resulting in a final valid sample of 1,712 students (valid response rate: 86.1%). The detailed enrollment and screening process is illustrated in the Participant Flow Diagram (Fig. 1).

### Measures (instruments)

### The Depression, Anxiety, and Stress Scales-21 (DASS-21)

Participants’ psychological distress over the preceding week was assessed using the validated Chinese version of the Depression, Anxiety, and Stress Scales-21 (DASS-21) [25, 27]. The DASS-21 is a 21-item self-report instrument comprising three seven-item subscales designed to measure the distinct states of depression, anxiety, and stress. Participants rated the extent to which they experienced each state on a 4-point Likert scale, ranging from 0 (“Did not apply to me at all”) to 3 (“Applied to me very much, or most of the time”). The total score for each of the three subscales (Depression, Anxiety, and Stress) was initially derived by summing the scores of the 7 items belonging to that specific dimension. As per standard scoring protocol, the sum of the scores for each subscale was multiplied by two to ensure comparability with the full DASS-42 version [31].

The severity of symptoms was classified into five categories (Normal, Mild, Moderate, Severe, Extremely Severe) based on the established cut-off scores, which are detailed in Table 1. The prevalence rates of depression, anxiety, and stress reported in this study were derived from these classifications. In the current sample, the DASS-21 demonstrated good internal consistency, with Cronbach’s α coefficients of 0.76 for the Stress subscale, 0.80 for the Anxiety subscale, and an acceptable coefficient of 0.72 for the Depression subscale.

**Table 1:** DASS-21 Severity Cut-off Scores

Category	Depression	Anxiety	Stress
Normal	0–9	0–7	0–14
Mild	10–13	8–9	15–18
Moderate	14–20	10–14	19–25
Severe	21–27	15–19	26–33
Extremely Severe	28+	20+	34+

Note: Scores are calculated by summing the relevant items and multiplying by two

### The Simplified Coping Style Questionnaire (SCSQ)

Coping strategies were evaluated using the Simplified Coping Style Questionnaire (SCSQ) [32]. This 20-item instrument is designed to assess individuals’ tendencies in managing stressful life events and is structured around two distinct dimensions: Positive Coping (12 items, e.g., “Look for the good in what has happened”) and Negative Coping (8 items, e.g., “Try to forget the whole thing”). Participants rated their use of each strategy on a 4-point scale from 0 (“Never”) to 3 (“Often”). Subscale scores for Positive Coping and Negative Coping were calculated as the mean score of the constituent items for each dimension. In the absence of a fixed clinical cut-off for the SCSQ, a higher mean score on a particular subscale relative to the sample average or the opposing subscale was used to identify the student’s predominant coping tendency. Higher scores on the respective subscales indicate a greater propensity to employ either positive or negative coping mechanisms. The SCSQ has demonstrated strong reliability and validity in Chinese populations. In this study, the internal consistency was excellent, with Cronbach’s α coefficients of 0.90 for the full scale, 0.89 for the Positive Coping subscale, and 0.78 for the Negative Coping subscale.

sadsaasd

### Socio-demographic and experiential questionnaire

A self-developed questionnaire was used to collect data on participants’ social background, life habits, and personal experiences. This questionnaire included items covering key variables such as age, gender, grade level, university region, and financial burden. To ensure full transparency, specific experiential variables were assessed and categorized as follows: Academic performance was measured via self-reported cumulative Grade Point Average (GPA) on a 5.0 scale. Because grading systems vary across Chinese universities, participants from institutions utilizing a 4.0 scale or a 100-point percentage system were provided with a standardized national conversion table within the survey instructions to convert their scores to the 5.0 scale, which were then categorized into four groups (2.0-2.5, 2.5–3.5, 3.5-4.0, 4.0–5.0). Self-reported physical health was assessed with a single item asking participants to rate their current overall physical condition, categorized as ‘Healthy’ or ‘Unwell’. Satisfaction with appearance was evaluated by asking students to rate their physical appearance as ‘Satisfactory’, ‘Average’, or ‘Dissatisfied’. Counseling experience was captured via a binary (Yes/No) question asking, “Have you ever utilized professional psychological counseling services?” Furthermore, the variable ‘excessive family interference’ was assessed with a single binary (Yes/No) item: “Do you feel your family interferes in your life excessively?” Full details of the variables collected are provided in Table 2.

**Table 2:** Sociodemographic and Baseline Characteristics of the Study Population (*N* = 1,712)

Variables	Frequency (*n*)	Percentage (%)
Geographical Region		
North	230	13.43
South	545	31.83
East	231	13.49
West	213	12.44
Central	493	28.80
Gender		
Male	719	42.00
Female	993	58.00
Academic Year		
1st	372	21.73
2nd	260	15.19
3rd	289	16.88
4th	366	21.38
5th	425	24.82
Marital Status		
In a relationship	430	25.12
Single	1,265	73.89
Married/Divorced	17	0.99
Home Location		
Rural	790	46.14
Urban	922	53.86
College GPA (out of 5.0)		
2.0–2.5	153	8.94
2.5–3.5	738	43.11
3.5–4.0	667	38.96
4.0–5.0	154	9.00
Dentistry as First-choice Major		
Yes	1,323	77.28
No	389	22.72
Financial Responsibility		
Yes	349	20.39
No	1,363	79.61
Excessive Family Interference		
Yes	295	17.23
No	1,417	82.77
Self-reported Physical Health		
Healthy	1,308	76.40
Unwell	404	23.60
Satisfaction with Appearance		
Satisfactory	573	33.47
Average	923	53.91
Dissatisfied	216	12.62
Counseling Experience		
Yes	338	19.74
No	1,374	80.26

Note: Data are presented as absolute frequencies (n) and percentages (%). Abbreviations: GPA, Grade Point Average. Percent totals may not equal 100% due to rounding

### Statistical analysis

**Fig. 2:**
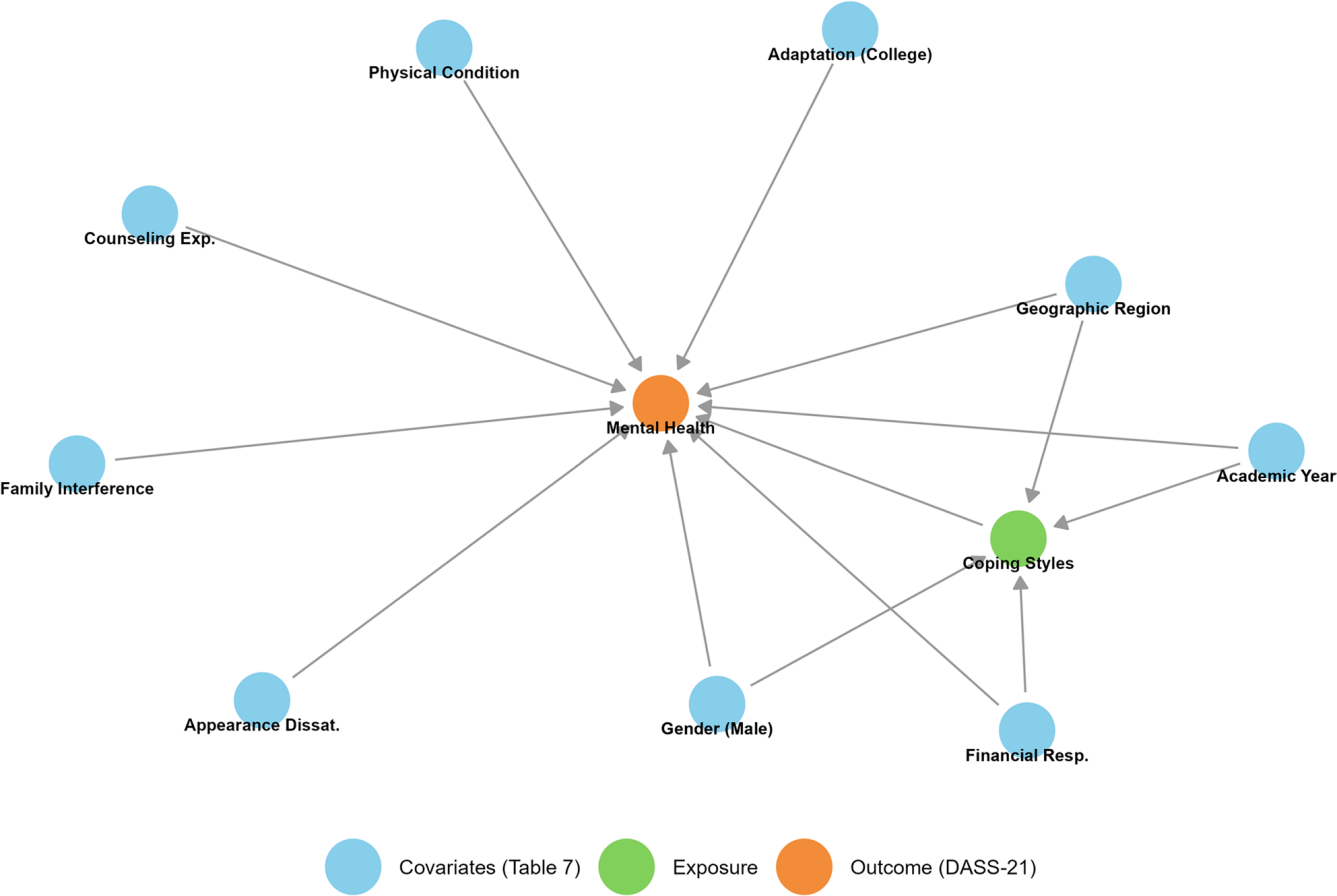
Directed Acyclic Graph (DAG) for Variable Selection. This revised DAG framework outlines the hypothesized causal pathways in full transparency. Coping Styles serve as the primary exposure; Mental Health (DASS-21) is the outcome. Academic Year, Gender (Male), Geographic Region, and Financial Responsibilities (Financial Resp.) are identified as antecedent confounders, hypothesized to influence both coping styles and mental health. All other individual variables included in the adjusted multivariable models—Physical Condition (unwell), Adaptation (College), Appearance Dissatisfaction (Appearance Dissat.), Family Interference, and Counseling Experience (Counseling Exp.)—are clearly delineated as experiential covariates with direct effects on mental health. All delineated variables are accounted for in the multivariable analysis to obtain adjusted estimates

To ensure the structural validity of our multivariable analyses, we developed a Directed Acyclic Graph (DAG) framework to inform variable selection and model adjustment (Fig. 2). In this framework, sociodemographic factors (e.g., gender, age, and geographical region) and academic stressors (e.g., academic year) were identified as antecedent variables. Coping styles (positive and negative) were conceptualized as the primary exposure variables associated with mental health outcomes. Sociodemographic characteristics were treated as potential confounders that could influence both the choice of coping mechanisms and the levels of psychological distress. Based on this DAG, we utilized a simultaneous entry method in our regression models to adjust for these confounders, thereby isolating the independent associations between coping styles, experiential factors, and DASS-21 scores. Statistical analyses were performed using Stata/MP 17.0 (StataCorp LLC, TX, USA). The survey platform’s mandatory response setting ensured zero item-level missing data, allowing for a complete-case analysis of 1,712 participants. Continuous variables were expressed as Mean ± SD, and categorical variables as frequencies (%). Data normality was assessed using Shapiro-Wilk tests and visual inspection of Q-Q plots; non-parametric Mann-Whitney U or Kruskal-Wallis H tests were applied where normality was violated, with post-hoc Tukey tests for multiple comparisons. Categorical associations were examined via Chi-square tests with Cramer’s V for effect size. Multivariable models were constructed using the “enter” method to identify factors independently associated with mental health. All sociodemographic factors and SCSQ coping dimensions were included simultaneously in the models to adjust for potential confounding and obtain independent estimates. The primary outcomes of this study were the levels of depression, anxiety, and stress among dental students. To ensure a comprehensive analysis, these outcomes were operationalized both as continuous subscale scores to capture the full spectrum of psychological distress and maximize statistical sensitivity in linear models, and as categorical severity levels (Normal to Extremely Severe) based on established DASS-21 cut-offs to provide clinically relevant prevalence data. Predictor variables included all variables collected in the sociodemographic questionnaire. Multiple linear regression was used for continuous scores, while multivariable ordinal logistic regression (ordered logit) models were specifically utilized for the ordinal outcome of DASS-21 severity categories (ranging from Normal to Extremely Severe). The outcome was not dichotomized. Prior to interpreting the multiple linear regression models, all relevant statistical assumptions were rigorously evaluated and met. Specifically, the linearity of continuous relationships was confirmed via partial regression plots; the independence of residuals was verified using the Durbin-Watson statistic (values approximating 2.00); homoscedasticity was assessed through visual inspection of scatterplots depicting standardized residuals against predicted values; and the normality of residuals was confirmed via histograms and normal Q-Q plots. Furthermore, prior to the interpretation of the ordinal logistic regression models, the proportional odds assumption was verified using the Brant test, which confirmed that the effects of the predictors were consistent across all thresholds of severity, thereby justifying the presentation of a single adjusted odds ratio (AOR) for each predictor. Multicollinearity was assessed using Variance Inflation Factors (VIF), with all values < 5.00. A two-tailed *p* < 0.05 was considered significant; however, given the exploratory nature and multiple comparisons, findings should be interpreted with caution regarding potential Type I errors.

## Result​

### Participant characteristics

The final study population consisted of 1,712 undergraduate dental students. As detailed in Table 2, the sample was predominantly female (*n* = 993, 58.00%) and represented a balanced distribution across all five academic years, with the largest cohorts being 5th-year (24.82%) and 1st-year (21.73%) students. Geographically, participants were recruited from 34 universities spanning five major regions of mainland China, with the highest concentrations in the Southern (31.83%) and Central (28.80%) regions.

To promote geographic diversity and mitigate over-representation from any single institution, the number of participants per institution was restricted to a range of 25 to 85, with no single university accounting for more than 5% of the total sample. (Supplementary Table S1). Regarding social and experiential backgrounds, the majority of respondents were single (73.8%), resided in urban areas (53.8%), and had enrolled in dentistry as their first-choice major (77.2%). Notably, a significant proportion of students reported facing financial responsibilities (20.4%), experiencing excessive family interference (17.2%), or perceiving their physical health as suboptimal (23.6%).

### Overall mental health status and coping styles

The overall psychological distress levels and coping style scores for the entire sample are summarized in Table 3. The mean total DASS-21 score was 12.86 (SD = 10.557). Among the three symptom domains, stress was the most pronounced (5.19 ± 4.094), followed by anxiety (3.89 ± 3.441) and depression (3.78 ± 3.749). When participants were classified by severity according to established cut-off scores, a significant proportion of students exhibited symptoms exceeding the normal range: 24.4% reported at-risk levels of stress, 46.1% for anxiety, and 36.0% for depression.

**Table 3:** Overall DASS-21 and Coping Style Scores of Dental Undergraduates (*N* = 1,712)

Variable	*N*	Mean ± SD	Min	Max
Psychological Distress (DASS-21)				
Total DASS-21 Score	1,712	12.86 ± 10.557	0	63
Stress Score	1,712	5.19 ± 4.094	0	21
Anxiety Score	1,712	3.89 ± 3.441	0	21
Depression Score	1,712	3.78 ± 3.749	0	21
Coping Styles (SCSQ)				
Positive Coping	1,712	21.37 ± 7.335	0	36
Negative Coping	1,712	9.79 ± 4.368	0	24

Note: *N* = 1,712. DASS-21 subscale scores were calculated by summing the relevant 7 items and multiplying by two to ensure comparability with the full 42-item scale. Abbreviations: *DASS-21 *21-item Depression Anxiety Stress Scales, *SCSQ *Simplified Coping Style Questionnaire, *SD * Standard Deviation

Regarding coping mechanisms, dental undergraduates scored higher on the positive coping subscale (Mean = 21.37, SD = 7.34) than on the negative coping subscale (Mean = 9.79, SD = 4.37) (Table 3). The detailed frequency and percentage distribution for each discrete score across both coping dimensions are provided in Supplementary Table S2.

### Group differences in mental health: bivariate analyses

Bivariate analyses revealed significant demographic and regional disparities in psychological distress among dental undergraduates. Regarding gender, males reported significantly higher mean scores across all DASS-21 subscales compared to females (Supplementary Table S3, *p* < 0.05). This trend was further reflected in the severity distribution; as shown in Table 4, a higher proportion of male students experienced moderate to severe levels of anxiety and depression compared to their female counterparts (*p* < 0.05).

**Table 4:** Crosstabulation of DASS-21 Subscale Severity Categories by Gender (*N* = 1,712)

Variable	Severity Level	Total *N* (%)	Male *n* (%)	Female *n* (%)	Chi-square (χ²)	*p*-value
Stress	Normal	1,295 (75.64)	516 (71.77)	779 (78.45)	12.05	0.017
	Mild	175 (10.22)	73 (10.15)	102 (10.27)		
	Moderate	171 (9.99)	96 (13.35)	75 (7.55)		
	Severe	42 (2.45)	23 (3.20)	19 (1.91)		
	Ext. Severe	29 (1.69)	11 (1.53)	18 (1.81)		
Anxiety	Normal	923 (53.91)	366 (50.90)	557 (56.09)	11.24	0.024
	Mild	323 (18.87)	136 (18.92)	187 (18.83)		
	Moderate	222 (12.97)	81 (11.27)	141 (14.20)		
	Severe	148 (8.64)	85 (11.82)	63 (6.34)		
	Ext. Severe	96 (5.61)	51 (7.09)	45 (4.53)		
Depression	Normal	1,095 (63.96)	432 (60.08)	663 (66.77)	18.95	< 0.001
	Mild	262 (15.30)	98 (13.63)	164 (16.52)		
	Moderate	272 (15.89)	141 (19.61)	131 (13.19)		
	Severe	41 (2.39)	24 (3.34)	17 (1.71)		
	Ext. Severe	42 (2.45)	24 (3.34)	18 (1.81)		

Note: Data represent frequencies (n) and row/column percentages (%). Statistical Test: P-values were calculated using the Pearson Chi-square test to compare the distribution of severity levels between genders. Abbreviations: Ext. Severe, Extremely Severe

**Fig. 3:**
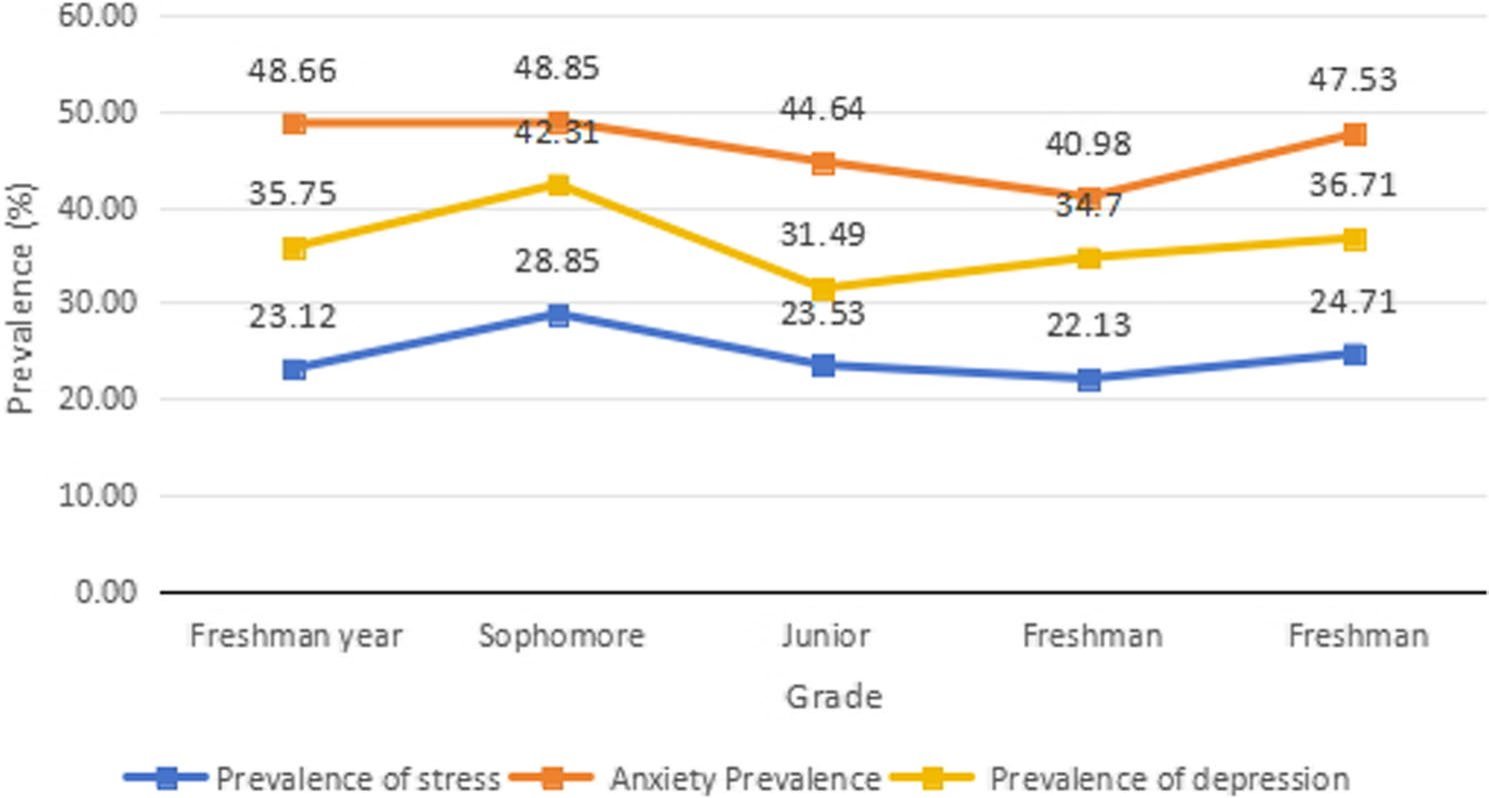
Depression-Anxiety-Stress faced by different school years

Psychological distress also varied significantly by academic year (Kruskal-Wallis H = 9.53, *p* = 0.049 for total score), with 5th-year students exhibiting the highest mean DASS-21 scores (Supplementary Table S4). This progression of stress, anxiety, and depression across the five-year curriculum is visualized in Fig. 3. Prevalence data mirrored this trend, with at-risk symptoms peaking in the 5th year and being lowest among 1st-year students (Supplementary Table S5).

Geographical location emerged as a particularly strong factor associated with mental health status. Students from the Western region exhibited significantly higher mean DASS-21 scores compared to those from all other regions (H = 84.91, *p* < 0.001; Table 5). The severity distribution confirmed this geographical disparity, with Western students reporting a dramatically higher prevalence of moderate-to-severe symptoms across all domains (*p* < 0.001; Supplementary Table S6). Other factors, including financial responsibility, suboptimal physical health, and dissatisfaction with personal appearance, were also significantly associated with elevated scores across all DASS-21 subscales (Supplementary Table S7).

**Table 5:** Geographic Disparities in DASS-21 Scores among Dental Undergraduates (*N* = 1,712)

Region	Total DASS-21 (Mean ± SD)	Stress (Mean ± SD)	Anxiety (Mean ± SD)	Depression (Mean ± SD)
North (*n* = 230)	10.85 ± 11.86	4.23 ± 4.36	3.28 ± 4.00	3.33 ± 3.98
South (*n* = 545)	13.54 ± 10.37	5.52 ± 4.12	4.04 ± 3.36	3.98 ± 3.80
East (*n* = 231)	11.48 ± 9.08	4.71 ± 3.44	3.59 ± 3.04	3.18 ± 3.32
West (*n* = 213)	17.81 ± 10.59	7.21 ± 4.18	5.12 ± 3.32	5.47 ± 3.85
Central (*n* = 493)	11.55 ± 10.04	4.61 ± 3.86	3.63 ± 3.35	3.31 ± 3.49
Kruskal-Wallis H	84.91	89.27	63.63	72.62
*p*-value	< 0.001	< 0.001	< 0.001	< 0.001

Note: Scores represent Mean ± Standard Deviation. Statistical Test: Comparison across regions was performed using the non-parametric Kruskal-Wallis H test due to the non-normal distribution of scores. Abbreviations: *DASS-21 *21-item Depression Anxiety Stress Scales, *SD *Standard Deviation

### Multivariable predictors of mental health and coping styles

In multivariable models designed to identify independent factors associated with psychological distress, coping styles emerged as consistent and powerful predictors. As shown in Table 6, the ordinal logistic regression analysis revealed that positive coping was a significant protective factor against increased symptom severity for stress (AOR = 0.90, 95% CI: 0.88–0.92, *p* < 0.001), anxiety (AOR = 0.92, 95% CI: 0.90–0.93, *p* < 0.001), and depression (AOR = 0.87, 95% CI: 0.85–0.88, *p* < 0.001). Conversely, negative coping strategies were significantly associated with an increased likelihood of higher symptom severity across all domains (*p* < 0.001), consistent with the transactional model of stress.

**Table 6:** Multivariable Ordered Logit Regression of Factors Associated with DASS-21 Severity Levels (*N* = 1,712)

Predictor	Stress AOR (95% CI)	*p*-value	Anxiety AOR (95% CI)	*p*-value	Depression AOR (95% CI)	*p*-value
Coping Style						
Positive Coping	0.90 (0.88, 0.92)	< 0.001	0.92 (0.90, 0.93)	< 0.001	0.87 (0.85, 0.88)	< 0.001
Negative Coping	1.18 (1.14, 1.21)	< 0.001	1.11 (1.07, 1.15)	< 0.001	1.17 (1.14, 1.21)	< 0.001
Physical/Experiential						
Physical Condition (Unwell)	4.04 (3.02, 5.40)	< 0.001	3.71 (2.79, 4.92)	< 0.001	4.44 (3.31, 5.95)	< 0.001
Adaptation (Poor/Average)	0.57 (0.44, 0.74)	< 0.001	0.63 (0.50, 0.79)	< 0.001	0.52 (0.41, 0.67)	< 0.001
Appearance (Dissatisfied)	0.60 (0.48, 0.75)	< 0.001	0.82 (0.68, 0.98)	0.027	0.69 (0.56, 0.84)	< 0.001
Family Interference (Yes)	0.59 (0.43, 0.82)	0.002	0.69 (0.51, 0.92)	0.012	0.82 (0.60, 1.13)	0.228
Sociodemographic						
Gender (Male)	0.89 (0.67, 1.18)	0.409	1.00 (0.79, 1.26)	0.993	0.94 (0.72, 1.21)	0.617
Geographic Region	0.99 (0.90, 1.09)	0.878	0.98 (0.91, 1.06)	0.625	0.93 (0.85, 1.01)	0.096
Academic Year	0.99 (0.90, 1.08)	0.812	0.98 (0.91, 1.05)	0.551	0.99 (0.91, 1.08)	0.847

Note: The model was adjusted for all variables listed simultaneously using the “enter” method. The outcome was not dichotomized; rather, an ordinal logistic regression was utilized across the five severity categories. 95% CIs for AORs were estimated based on standard errors. Abbreviations: *AOR *Adjusted Odds Ratio, *CI *Confidence Interval

When examining predictors of the continuous DASS-21 total and subscale scores using multiple linear regression (Table 7), self-perceived physical condition emerged as the strongest predictor of overall psychological distress (β = 0.37, 95% CI: 0.32 to 0.41, *p* < 0.001). Other significant experiential factors included dissatisfaction with personal appearance (β = -0.13, 95% CI: -0.18 to -0.09, *p* < 0.001) and difficulties adapting to college life (β = -0.15, 95% CI: -0.20 to -0.10, *p* < 0.001). Additionally, excessive family interference (β = -0.08, 95% CI: -0.13 to -0.04, *p* < 0.001), financial responsibility, and a history of counseling experience were independently associated with higher scores.

**Table 7:** Multiple Linear Regression Analysis of Factors Associated with Continuous DASS-21 Scores (*N* = 1,712)

Variable	Total Scoreβ (95% CI)	*p*-value	Stress Scoreβ (95% CI)	*p*-value	Anxiety Scoreβ (95% CI)	*p*-value	Depression Scoreβ (95% CI)	*p*-value
Coping Styles								
Positive Coping	-0.22 (-0.26, -0.17)	< 0.001	-0.19 (-0.24, -0.14)	< 0.001	-0.19 (-0.24, -0.15)	< 0.001	-0.22 (-0.27, -0.18)	< 0.001
Negative Coping	0.14 (0.10, 0.19)	< 0.001	0.12 (0.08, 0.17)	< 0.001	0.11 (0.06, 0.16)	< 0.001	0.15 (0.11, 0.20)	< 0.001
Core Predictors								
Physical Condition	0.37 (0.32, 0.41)	< 0.001	0.35 (0.31, 0.40)	< 0.001	0.32 (0.27, 0.37)	< 0.001	0.35 (0.30, 0.39)	< 0.001
Adaptation (College)	-0.15 (-0.20, -0.10)	< 0.001	-0.13 (-0.18, -0.09)	< 0.001	-0.13 (-0.18, -0.09)	< 0.001	-0.15 (-0.20, -0.11)	< 0.001
Appearance Dissat.	-0.13 (-0.18, -0.09)	< 0.001	-0.13 (-0.18, -0.08)	< 0.001	-0.11 (-0.16, -0.07)	< 0.001	-0.13 (-0.17, -0.08)	< 0.001
Family Interference	-0.08 (-0.13, -0.04)	< 0.001	-0.09 (-0.13, -0.04)	< 0.001	-0.09 (-0.13, -0.04)	< 0.001	-0.06 (-0.10, -0.02)	0.004
Counseling Exp.	-0.06 (-0.11, -0.02)	0.008	-0.07 (-0.11, -0.02)	0.009	-0.06 (-0.10, -0.01)	0.011	-0.05 (-0.09, -0.01)	0.015
Financial Resp.	-0.07 (-0.11, -0.03)	< 0.001	-0.07 (-0.12, -0.02)	0.007	-0.05 (-0.10, -0.01)	0.018	-0.09 (-0.14, -0.05)	< 0.001
Sociodemographics								
Gender (Male)	-0.04 (-0.08, 0.01)	0.091	-0.02 (-0.06, 0.03)	0.469	-0.03 (-0.08, 0.01)	0.161	-0.06 (-0.10, -0.01)	0.010
Geographic Region	-0.01 (-0.05, 0.03)	0.604	-0.01 (-0.05, 0.04)	0.724	0.00 (-0.04, 0.03)	0.956	-0.06 (-0.17, 0.05)	0.316

Note: “β” represents the standardized regression coefficient. 95% CIs were constructed based on standard errors. The multicollinearity of all predictors was assessed via Variance Inflation Factor (VIF), with all values below 2.0. Abbreviations: *CI *Confidence Interval, β, Standardized Regression Coefficient; Counseling Exp., Counseling Experience; Financial Resp., Financial Responsibility

## Discussion

To our knowledge, this is the first nationwide, multi-regional investigation of depression, anxiety, stress, and coping styles specifically among undergraduate dental students in China. By surveying 1,712 participants from 34 universities across the country, our study addresses a significant gap in the literature, which has previously been characterized by single-institution reports or studies focusing on the broader medical student population. Our findings not only provide a comprehensive baseline of the mental health landscape for Chinese dental undergraduates but also uncover crucial regional, academic-stage, and gender-specific variations, offering a nuanced foundation for developing targeted support and educational reforms.

Intensifying competition for education and employment in China has exacerbated psychological pressure among university students [33, 34], leading to a rise in mental health issues. While a national mental health survey highlighted significant psychological symptoms among medical students [18], our study offers dental-specific data, revealing a concerning burden. For instance, one study reporting depression, anxiety, and stress prevalence rates as high as 28.8%, 32.9%, and 17.9%, respectively, among graduating-year students [16]. These figures underscore the unique and demanding nature of the dental curriculum, which is characterized by a heavy academic load, intense examination pressure, and the early onset of clinical practice demands [19, 35–37]. This aligns with a systematic review that identified self-efficacy concerns, performance pressures, heavy workloads, and challenging faculty/patient interactions as major stressors in dental education [38].

Our finding that psychological problems are more prominent among students in Western China aligns with our hypothesis regarding regional disparities. We hypothesize that this may be linked to regional imbalances in economic development, educational resources, and healthcare infrastructure. Official reports confirm that Western China lags behind the more developed eastern regions in terms of economic output, household income, and access to quality health and education services [39, 40]. These socioeconomic disadvantages could translate into greater financial pressure and reduced social support for students, exacerbating their mental health challenges. This interpretation, while plausible and supported by macro-level data, requires validation through future region-specific studies focusing on dental students.

The stage of study also profoundly influences mental health. Our results, which show psychological distress increasing with academic seniority, are consistent with longitudinal findings from European dental schools [27]. The peak prevalence of stress, anxiety, and depression in fifth-year students highlights the immense pressure associated with final examinations, clinical competency assessments, and career uncertainty. Stress prevalence peaked in the junior and senior years, likely corresponding to the most intensive clinical training phases and employment-seeking pressures. Anxiety followed a distinct “U-shaped” curve, peaking in the first and fifth years, reflecting the psychological vulnerabilities during initial adaptation and the final transition-to-practice periods.

Contrary to most studies that report higher psychological distress in females [16, 36], our study found that male students exhibited significantly higher DASS-21 total and subscale scores. While this is an atypical finding, it is not unprecedented. Some research has indicated that male students may experience greater difficulties in specific domains, such as emotional exhaustion and burnout [41]. This may point to a more complex interplay between gender roles, help-seeking behaviors, and the expression of psychological distress in the Chinese cultural context. One possible explanation is that traditional norms of masculinity, which emphasize emotional control and stoicism, may discourage male students from acknowledging or seeking help for mental health struggles until they become severe [42, 43]. This reluctance, driven by the social pressure to appear strong and self-reliant, could lead to an accumulation of stress, resulting in higher reported symptom severity when finally measured anonymously. Furthermore, male students in China often face intense and specific pressures related to societal and familial expectations to achieve exceptional academic and career success, as they are traditionally viewed as future primary providers [44]. This burden, coupled with a cultural hesitancy to express vulnerability, may manifest as higher levels of stress and anxiety compared to their female counterparts, who may experience different societal pressures. Thus, our findings may not reflect a higher overall prevalence of mental health issues among males, but rather a greater severity of symptoms driven by unique cultural stressors and barriers to care. This discrepancy certainly warrants further mixed-methods investigation, such as combining longitudinal surveys with in-depth qualitative interviews to specifically explore how traditional masculine norms and help-seeking barriers influence male dental students’ stress appraisal.

Crucially, our study confirms the powerful moderating role of coping styles. Adaptive coping strategies, such as seeking social support and positive reframing, were strongly associated with better mental health outcomes, while negative coping styles (e.g., substance use, avoidance) were linked to increased psychological distress. This reinforces the importance of psychoeducation and targeted interventions to foster adaptive coping mechanisms [45–48]. Since some students resort to maladaptive behaviors like medication misuse to manage stress [49], it is vital that dental education institutions implement proactive support systems.

The significant negative effect of appearance satisfaction on mental health in our models, second only to physical health, underscores the substantial impact of body image in the modern era. This finding resonates with a growing body of literature linking heavy social media use to heightened appearance anxiety and depression among young adults, as platforms often promote idealized and unattainable beauty standards [50, 51]. The pressure to maintain a certain image, combined with academic perfectionism, may create a potent source of stress for dental students [52, 53].

However, this study has several limitations. First, its cross-sectional design precludes any inference of causality. Second, the use of an online convenience sampling method may introduce self-selection bias, as students with more pronounced mental health concerns might be more or less likely to participate, and it also risks social desirability bias in responses. Most importantly, while our substantial sample size permitted robust multivariable modeling, the use of convenience and snowball sampling techniques implies that true national representativeness was not achieved. Consequently, our prevalence data should be interpreted as reflecting the characteristics of this specific, albeit geographically diverse, cohort rather than absolute national epidemiological rates. The lack of a reported response rate further limits the generalizability of our findings. Third, our sample had an uneven regional distribution, with a smaller proportion of participants (12.4%) from Western China, which may affect the robustness of regional comparisons. Finally, unmeasured confounding variables, such as personality traits, prior mental health history, or specific curriculum details, may have influenced the outcomes.

Therefore, future research should prioritize longitudinal studies to track the trajectory of mental health from matriculation to graduation and beyond. There is also a clear need for intervention-based research to design and evaluate the effectiveness of tailored support programs, such as stress management workshops, peer-mentoring systems, and accessible counseling services integrated within dental schools. Further qualitative studies could provide deeper insights into the lived experiences and specific stressors faced by students, particularly those in underrepresented regions like Western China. Lastly, investigating the complex interplay between academic culture, social media exposure, and mental well-being would be a valuable avenue for future inquiry. In conclusion, in addition to imparting professional knowledge, dental educators must prioritize students’ physical and mental health. Given the inherently demanding nature of dental education, greater attention to students’ psychological well-being is imperative. Universities and their administrative departments must enhance their focus on student mental health and bolster the provision of comprehensive social support [54, 55].

## Conclusion

Chinese dental undergraduates experience a substantial burden of psychological distress, characterized primarily by anxiety and depression. This distress is disproportionately distributed, with students in Western China and those reporting suboptimal physical health emerging as particularly vulnerable populations. Crucially, coping styles play a vital moderating role: positive coping strategies act as robust buffers against mental health challenges, whereas negative coping, body image dissatisfaction, and poor college adaptation significantly exacerbate psychological burden. Dental education institutions must move beyond purely academic training to implement targeted, evidence-based psychological support systems. Prioritizing wellness programs that foster adaptive coping skills, alongside specific interventions for geographically and physically vulnerable cohorts, is essential for safeguarding the mental well-being of future dental professionals.

## Supplementary Information

Supplementary Material 1.

## Data Availability

The datasets used and analysed during the current study available from the corresponding author on reasonable request.
