# Supplementary material for: A survey on stress, anxiety, depression and coping styles in dental undergraduates from 34 universities in China
Source: BMC Med Educ. 2026 May 9;26:1055. doi: 10.1186/s12909-026-09371-9 (PMC13326217; doi:10.1186/s12909-026-09371-9)
Supplement: Supplementary file 1 — Supplementary Material 1. [file 12909_2026_9371_MOESM1_ESM.docx]

**Supplementary Table S1: Institutional and Grade-level Distribution of Participants**

| **Region** | **University** | **1st Year** | **2nd Year** | **3rd Year** | **4th Year** | **5th Year** | **Total** |
| --- | --- | --- | --- | --- | --- | --- | --- |
| North | Peking University | 8 | 7 | 11 | 9 | 10 | 45 |
| (n=230) | Capital Medical University | 11 | 9 | 6 | 12 | 11 | 49 |
|  | Tianjin Medical University | 7 | 8 | 6 | 11 | 9 | 41 |
|  | Hebei Medical University | 9 | 7 | 7 | 9 | 13 | 45 |
|  | China Medical University | 12 | 10 | 8 | 8 | 12 | 50 |
| South | Sun Yat-sen University | 13 | 10 | 7 | 7 | 13 | 50 |
| (n=545) | Guangzhou Medical University | 19 | 9 | 7 | 10 | 12 | 57 |
|  | Southern Medical University | 13 | 5 | 10 | 8 | 17 | 53 |
|  | Guangxi Medical University | 9 | 11 | 10 | 13 | 20 | 63 |
|  | Fujian Medical University | 14 | 8 | 8 | 16 | 12 | 58 |
|  | Hainan Medical University | 16 | 4 | 14 | 14 | 10 | 58 |
|  | Shantou University | 15 | 11 | 9 | 13 | 19 | 67 |
|  | Shenzhen University | 11 | 12 | 15 | 13 | 19 | 70 |
|  | Jinan University | 10 | 14 | 14 | 11 | 20 | 69 |
| East | Shanghai Jiao Tong University | 7 | 4 | 5 | 9 | 12 | 37 |
| (n=231) | Nanjing Medical University | 9 | 11 | 8 | 11 | 16 | 55 |
|  | Zhejiang University | 9 | 6 | 8 | 18 | 14 | 55 |
|  | Shandong University | 11 | 7 | 9 | 7 | 10 | 44 |
|  | Anhui Medical University | 8 | 6 | 7 | 9 | 10 | 40 |
| Western | Sichuan University | 12 | 6 | 7 | 9 | 12 | 46 |
| (n=213) | Chongqing Medical University | 6 | 5 | 6 | 7 | 10 | 34 |
|  | Xi'an Jiaotong University | 12 | 8 | 6 | 8 | 8 | 42 |
|  | Xinjiang Medical University | 8 | 7 | 7 | 12 | 8 | 42 |
|  | Lanzhou University | 9 | 7 | 11 | 11 | 11 | 49 |
| Central | Wuhan University | 11 | 6 | 7 | 12 | 11 | 47 |
| (n=493) | Huazhong Univ. of Sci. & Tech. | 15 | 9 | 10 | 11 | 9 | 54 |
|  | Central South University | 8 | 7 | 7 | 9 | 13 | 44 |
|  | Zhengzhou University | 8 | 8 | 7 | 9 | 14 | 46 |
|  | Hubei University of Medicine | 13 | 6 | 8 | 11 | 6 | 44 |
|  | Henan University | 11 | 7 | 4 | 11 | 11 | 44 |
|  | Hunan Normal University | 14 | 6 | 7 | 12 | 16 | 55 |
|  | Nanchang University | 13 | 7 | 12 | 11 | 10 | 53 |
|  | Shanxi Medical University | 13 | 7 | 13 | 10 | 11 | 54 |
|  | Gannan Medical University | 8 | 5 | 8 | 15 | 16 | 52 |
| Total |  | 372 | 260 | 289 | 366 | 425 | 1,712 |

Note: N = 1,712. This table provides the raw distribution of participants across all 34 participating institutions. Classification: Regions (North, South, East, West, Central) were defined according to the official geographical division of mainland China. The purposive sampling strategy ensured that no single institution contributed more than 5% of the total sample size to minimize institutional bias**.**

**Supplementary Table 2. Distribution of positive and negative coping scores among participants**

| **Score** | **Positive Coping n (%)** | **Negative Coping n (%)** | **Score** | **Positive Coping n (%)** | **Negative Coping n (%)** |
| --- | --- | --- | --- | --- | --- |
| 0 | 21 (1.23) | 18 (1.05) | 19 | 64 (3.74) | 16 (0.93) |
| 1 | 1 (0.06) | 10 (0.58) | 20 | 78 (4.56) | 6 (0.35) |
| 2 | 6 (0.35) | 23 (1.34) | 21 | 84 (4.91) | 10 (0.58) |
| 3 | 5 (0.29) | 27 (1.58) | 22 | 86 (5.02) | 5 (0.29) |
| 4 | 5 (0.29) | 50 (2.92) | 23 | 115 (6.72) | 31 (1.81) |
| 5 | 1 (0.06) | 99 (5.78) | 24 | 121 (7.07) | 18 (1.05) |
| 6 | 1 (0.06) | 120 (7.01) | 25 | 86 (5.02) | - |
| 7 | 2 (0.12) | 169 (9.87) | 26 | 61 (3.56) | - |
| 8 | 8 (0.47) | 204 (11.92) | 27 | 73 (4.26) | - |
| 9 | 11 (0.64) | 223 (13.03) | 28 | 49 (2.86) | - |
| 10 | 16 (0.93) | 133 (7.77) | 29 | 37 (2.16) | - |
| 11 | 36 (2.10) | 118 (6.89) | 30 | 36 (2.10) | - |
| 12 | 79 (4.61) | 125 (7.30) | 31 | 42 (2.45) | - |
| 13 | 50 (2.92) | 113 (6.60) | 32 | 32 (1.87) | - |
| 14 | 62 (3.62) | 53 (3.10) | 33 | 24 (1.40) | - |
| 15 | 71 (4.15) | 47 (2.75) | 34 | 29 (1.69) | - |
| 16 | 96 (5.61) | 61 (3.56) | 35 | 15 (0.88) | - |
| 17 | 55 (3.21) | 31 (1.81) | 36 | 52 (3.04) | - |
| 18 | 83 (4.85) | 20 (1.17) |  |  |  |

Note: Data represent frequencies (n) and percentages (%). SCSQ dimensions are scored on different ranges (Positive: 0–36; Negative: 0–24). Abbreviations: SCSQ, Simplified Coping Style Questionnaire.

**Supplementary Table S3. Comparison of DASS-21 Scores between Genders (N = 1,712)**

| **Variable** | **Male (n = 719) Mean ± SD** | **Female (n = 993) Mean ± SD** | **Mann-Whitney U** | **p-value** |
| --- | --- | --- | --- | --- |
| DASS-21 Total | 13.90 ± 11.37 | 12.11 ± 9.86 | 329,766.50 | 0.003 |
| Stress | 5.51 ± 4.34 | 4.96 ± 3.89 | 334,115.50 | 0.023 |
| Anxiety | 4.18 ± 3.72 | 3.68 ± 3.21 | 334,161.00 | 0.023 |
| Depression | 4.20 ± 3.98 | 3.47 ± 3.54 | 321,148.00 | < 0.001 |

Note: Data are presented as Mean ± Standard Deviation. Statistical Test: P-values were calculated using the non-parametric Mann-Whitney U test due to the non-normal distribution of psychological scores. Abbreviations: DASS-21, 21-item Depression Anxiety Stress Scales; SD, Standard Deviation

**Supplementary Table S4. Comparison of DASS-21 Scores among Different Academic Years (N = 1,712)**

| **Academic Year** | **Total Score (Mean ± SD)** | **Stress (Mean ± SD)** | **Anxiety (Mean ± SD)** | **Depression (Mean ± SD)** |
| --- | --- | --- | --- | --- |
| 1st-year (n=372) | 13.15 ± 9.59 | 5.29 ± 3.82 | 4.17 ± 3.13 | 3.69 ± 3.42 |
| 2nd-year (n=260) | 13.68 ± 10.29 | 5.50 ± 4.03 | 4.06 ± 3.25 | 4.12 ± 3.71 |
| 3rd-year (n=289) | 12.39 ± 10.01 | 5.00 ± 3.97 | 3.74 ± 3.27 | 3.64 ± 3.57 |
| 4th-year (n=366) | 11.68 ± 10.13 | 4.84 ± 4.01 | 3.39 ± 3.28 | 3.46 ± 3.62 |
| 5th-year (n=425) | 13.44 ± 12.08 | 5.34 ± 4.49 | 4.09 ± 3.99 | 4.02 ± 4.24 |
| Kruskal-Wallis H | 9.53 | 6.36 | 18.95 | 6.56 |
| p-value | 0.049 | 0.174 | < 0.001 | 0.161 |

Note: Scores represent Mean ± Standard Deviation. Statistical Test: Differences among the five academic years were assessed using the Kruskal-Wallis H test. Abbreviations: SD, Standard Deviation.

**Supplementary Table S5. Prevalence of DASS-21 Severity Levels (%) Categorized by Academic Year and Gender (N = 1,712)**


| **Variable / Severity** | **Year 1 (%) M / F** | **Year 2 (%) M / F** | **Year 3 (%) M / F** | **Year 4 (%) M / F** | **Year 5 (%) M / F** | **χ2 (p-value)** |
| --- | --- | --- | --- | --- | --- | --- |
| Stress |  |  |  |  |  | 23.01 (< 0.001) |
| Normal | 68.00 / 84.00 | 65.70 / 74.70 | 69.00 / 78.00 | 72.00 / 80.00 | 58.00 / 68.00 |  |
| Mild | 7.90 / 8.20 | 11.80 / 14.60 | 10.00 / 12.00 | 9.00 / 10.00 | 15.00 / 16.00 |  |
| Moderate | 21.80 / 5.20 | 16.60 / 7.60 | 16.00 / 7.00 | 14.00 / 7.00 | 18.00 / 10.00 |  |
| Severe | 1.70 / 2.10 | 3.90 / 2.50 | 3.00 / 2.00 | 3.00 / 2.00 | 6.00 / 4.00 |  |
| Ext. Severe | 0.60 / 0.50 | 2.00 / 0.60 | 2.00 / 1.00 | 2.00 / 1.00 | 3.00 / 2.00 |  |
| Anxiety |  |  |  |  |  | 29.77 (< 0.001) |
| Normal | 42.10 / 59.80 | 45.10 / 55.10 | 48.00 / 58.00 | 52.00 / 62.00 | 40.00 / 50.00 |  |
| Mild | 18.60 / 17.50 | 16.70 / 20.90 | 18.00 / 19.00 | 16.00 / 18.00 | 20.00 / 22.00 |  |
| Moderate | 11.20 / 13.90 | 15.70 / 13.90 | 14.00 / 12.00 | 14.00 / 10.00 | 18.00 / 15.00 |  |
| Severe | 24.20 / 5.20 | 13.70 / 5.70 | 13.00 / 6.00 | 12.00 / 6.00 | 14.00 / 8.00 |  |
| Ext. Severe | 3.90 / 3.60 | 8.80 / 4.40 | 7.00 / 5.00 | 6.00 / 4.00 | 8.00 / 5.00 |  |
| Depression |  |  |  |  |  | 30.09 (< 0.001) |
| Normal | 55.10 / 72.70 | 47.10 / 64.60 | 50.00 / 66.00 | 54.00 / 70.00 | 42.00 / 60.00 |  |
| Mild | 10.10 / 16.00 | 21.60 / 16.40 | 20.00 / 15.00 | 18.00 / 14.00 | 22.00 / 18.00 |  |
| Moderate | 31.40 / 9.80 | 21.60 / 15.20 | 20.00 / 14.00 | 19.00 / 12.00 | 24.00 / 16.00 |  |
| Severe | 2.80 / 1.00 | 4.80 / 2.50 | 5.00 / 3.00 | 5.00 / 2.00 | 7.00 / 4.00 |  |
| Ext. Severe | 0.60 / 0.50 | 4.90 / 1.30 | 5.00 / 2.00 | 4.00 / 2.00 | 5.00 / 2.00 |  |

Note: M = Male, F = Female. Data represent the percentage of students within each gender-year subgroup. Statistical Test: Pearson’s Chi-square test was used to evaluate the associations between severity distribution, gender, and academic year. Abbreviations: Ext. Severe, Extremely Severe.

**Supplementary Table S6. Prevalence of DASS-21 Severity Levels (%) by Geographic Region and Gender (N = 1,712)**


| **Variable / Severity** | **North (%) M / F** | **South (%) M / F** | **East (%) M / F** | **West (%) M / F** | **Central (%) M / F** | **χ2 (p-value)** |
| --- | --- | --- | --- | --- | --- | --- |
| Stress |  |  |  |  |  | 13.05 (0.011) |
| Normal | 78.20 / 88.10 | 72.00 / 73.90 | 75.30 / 80.10 | 64.20 / 69.80 | 76.50 / 79.40 |  |
| Mild | 6.90 / 4.90 | 14.00 / 10.80 | 10.10 / 11.20 | 18.10 / 15.40 | 11.20 / 9.80 |  |
| Moderate | 4.60 / 6.30 | 10.00 / 8.10 | 11.20 / 6.70 | 13.50 / 10.20 | 9.40 / 8.20 |  |
| Severe | 3.40 / 0.00 | 3.20 / 3.70 | 2.50 / 1.50 | 3.40 / 3.10 | 2.10 / 2.00 |  |
| Ext. Severe | 6.90 / 0.70 | 0.80 / 3.40 | 0.90 / 0.50 | 0.80 / 1.50 | 0.80 / 0.60 |  |
| Anxiety |  |  |  |  |  | 12.47 (0.014) |
| Normal | 66.70 / 60.80 | 52.00 / 50.50 | 58.20 / 62.40 | 48.20 / 51.50 | 55.40 / 58.20 |  |
| Mild | 9.20 / 17.50 | 20.40 / 21.70 | 18.50 / 16.90 | 22.10 / 20.40 | 19.20 / 18.50 |  |
| Moderate | 11.50 / 13.30 | 12.00 / 14.90 | 12.40 / 13.10 | 15.40 / 16.20 | 12.80 / 11.40 |  |
| Severe | 1.10 / 5.60 | 8.80 / 7.10 | 7.40 / 5.10 | 9.80 / 8.40 | 8.50 / 7.80 |  |
| Ext. Severe | 11.50 / 2.80 | 6.80 / 5.80 | 3.50 / 2.50 | 4.50 / 3.50 | 4.10 / 4.10 |  |
| Depression |  |  |  |  |  | 8.64 (0.071) |
| Normal | 67.80 / 70.60 | 62.00 / 60.00 | 65.40 / 68.20 | 58.40 / 62.10 | 64.20 / 65.10 |  |
| Mild | 11.50 / 13.30 | 12.40 / 22.70 | 14.20 / 15.10 | 18.50 / 17.20 | 15.10 / 16.20 |  |
| Moderate | 10.30 / 14.00 | 18.80 / 13.20 | 16.10 / 14.20 | 19.40 / 16.50 | 17.20 / 15.40 |  |
| Severe | 3.40 / 1.40 | 3.60 / 2.00 | 2.50 / 1.80 | 2.10 / 2.80 | 2.10 / 1.80 |  |
| Ext. Severe | 6.90 / 0.70 | 3.20 / 2.00 | 1.80 / 0.70 | 1.60 / 1.40 | 1.40 / 1.50 |  |

Note: M = Male, F = Female. Data represent the percentage (%) of participants within each subgroup. Statistical Test: Pearson’s Chi-square test was used to examine the associations between psychological severity, geographic region, and gender. Abbreviations: Ext. Severe, Extremely Severe.

**Supplementary Table S7.** Bivariate Analysis of DASS-21 Subscale Scores across Sociodemographic and Experiential Variables (N = 1,712)


| **Variables** | **Stress (Mean ± SD)** | **p-value** | **Anxiety (Mean ± SD)** | **p-value** | **Depression (Mean ± SD)** | **p-value** |
| --- | --- | --- | --- | --- | --- | --- |
| Marital Status |  | < 0.001 |  | < 0.001 |  | < 0.001 |
| In a relationship | 4.98 ± 4.13 |  | 3.73 ± 3.51 |  | 3.50 ± 3.62 |  |
| Single | 5.18 ± 3.98 |  | 3.87 ± 3.27 |  | 3.77 ± 3.64 |  |
| Married/Divorced | 11.15 ± 7.09 |  | 10.41 ± 7.27 |  | 11.63 ± 6.45 |  |
| Home Location |  | 0.074 |  | 0.009 |  | 0.055 |
| Rural | 5.34 ± 4.17 |  | 4.12 ± 3.52 |  | 3.93 ± 3.67 |  |
| Urban | 5.06 ± 4.03 |  | 3.70 ± 3.37 |  | 3.64 ± 3.81 |  |
| Financial Responsibility |  | < 0.001 |  | < 0.001 |  | < 0.001 |
| Yes | 6.15 ± 4.88 |  | 4.67 ± 4.18 |  | 4.90 ± 4.76 |  |
| No | 4.94 ± 3.83 |  | 3.69 ± 3.19 |  | 3.49 ± 3.39 |  |
| Excessive Interference |  | < 0.001 |  | < 0.001 |  | < 0.001 |
| Yes | 6.63 ± 4.44 |  | 5.10 ± 4.03 |  | 4.97 ± 4.25 |  |
| No | 4.89 ± 3.95 |  | 3.64 ± 3.25 |  | 3.53 ± 3.59 |  |
| University Life Adaptation |  | < 0.001 |  | < 0.001 |  | < 0.001 |
| Poor | 8.74 ± 6.25 |  | 7.83 ± 6.12 |  | 7.43 ± 6.16 |  |
| Average | 6.35 ± 4.26 |  | 4.77 ± 3.46 |  | 4.93 ± 3.86 |  |
| Good | 4.49 ± 3.76 |  | 3.34 ± 3.20 |  | 3.08 ± 3.41 |  |
| Appearance Satisfaction |  | < 0.001 |  | < 0.001 |  | < 0.001 |
| Dissatisfied | 7.81 ± 4.91 |  | 5.98 ± 4.07 |  | 6.26 ± 4.63 |  |
| Average | 5.23 ± 3.74 |  | 3.90 ± 3.20 |  | 3.80 ± 3.41 |  |
| Satisfactory | 4.13 ± 3.85 |  | 3.10 ± 3.23 |  | 2.81 ± 3.46 |  |
| Counseling Experience |  | 0.008 |  | 0.016 |  | 0.025 |
| Yes | 5.79 ± 4.69 |  | 4.30 ± 4.05 |  | 4.20 ± 4.56 |  |
| No | 5.04 ± 3.92 |  | 3.79 ± 3.27 |  | 3.67 ± 3.52 |  |
| Physical Condition |  | < 0.001 |  | < 0.001 |  | < 0.001 |
| Unwell | 8.25 ± 4.37 |  | 6.28 ± 3.86 |  | 6.66 ± 4.16 |  |
| Healthy | 4.24 ± 3.50 |  | 3.16 ± 2.93 |  | 2.89 ± 3.11 |  |
| Lifestyle Habits |  |  |  |  |  |  |
| Smoking (Yes) | 6.41 ± 5.46 | 0.019 | 5.30 ± 5.24 | 0.009 | 5.30 ± 5.03 | 0.009 |
| Drinking (Yes) | 5.97 ± 4.78 | 0.007 | 4.80 ± 4.16 | < 0.001 | 4.64 ± 4.34 | < 0.001 |

Note: Data are presented as Mean ± Standard Deviation. Statistical Test: P-values were determined using independent t-tests or one-way ANOVA for variables with more than two groups. Non-parametric alternatives were used where normality was violated. Abbreviations: DASS-21, 21-item Depression Anxiety Stress Scales; SD, Standard Deviation.
